# *In vivo* imaging of uterine cervix with a Mueller polarimetric colposcope

**DOI:** 10.1038/s41598-017-02645-9

**Published:** 2017-06-01

**Authors:** Jérémy Vizet, Jean Rehbinder, Stanislas Deby, Stéphane Roussel, André Nazac, Ranya Soufan, Catherine Genestie, Christine Haie-Meder, Hervé Fernandez, François Moreau, Angelo Pierangelo

**Affiliations:** 1 LPICM, CNRS, Ecole Polytechnique, Université Paris Saclay, Palaiseau, France; 2 Department of Obstetrics and Gynecology, University Hospital Brugmann, Université Libre de Bruxelles, Bruxelles, Belgium; 3 Institut Gustave Roussy, Service d’anatomie pathologique gynécologique, Villejuif, France; 4 Institut Gustave Roussy, Service de Curiethérapie, Villejuif, France; 5 Service de Gynécologie Obstétrique, CHU de Bicêtre AP-HP, Le Kremlin-Bicêtre, France

## Abstract

Mueller polarimetric imaging enables the detection and quantification of modifications of the collagen fibers in the uterine cervix due to the development of a precancerous lesion. This information is not accessible through the use of the classic colposcope, a low magnification microscope used in current practice for cervical cancer screening. However, the *in vivo* application of Mueller polarimetric imaging poses an instrumental challenge: the device should be sufficiently compact, while still being able to perform fast and accurate acquisition of Mueller matrices in real-world conditions. In this study, the first wide field Mueller Polarimetric Colposcope (MPC) for the *in vivo* analysis of uterine cervix is presented. The MPC has been fabricated by grafting a miniaturized Mueller polarimetric imager on a classic colposcope. This new imaging tool performs the fast acquisition of Mueller polarimetric images, thus eliminating any blurring effects due to patient movements. It can be easily used by a practitioner with little change to their existing practice. Finally, the MPC was tested *in vivo* on a number of patients in the field.

## Introduction

In recent years, optical polarimetry has received considerable attention as a medical diagnostic tool, as techniques based on this method provide additional information compared to intensity-based measurements. Polarimetric imaging is very promising for biomedical applications for several reasons: (i) it exploits the polarization of light, which is sensitive to morphological changes in the structure of tissues on a microscopic scale; (ii) it provides wide field images (up to 20 cm^2^) for the analysis of large areas in biomedical applications; (iii) it is a non-invasive technique, enabling one to obtain images of tissue without contact and without the use of chemical products; (iv) it can be easily implemented using conventional, low-cost white light sources such as LEDs or halogen lamps which are innocuous for patients. Recent studies have shown the great potential of polarimetric imaging in detecting pathological areas on a striking variety of tissues^1^ such as skin^2–4^, intestine^5^, colon^6–10^, and rectum^11^. This technique can be implemented in several ways, ranging from the relatively simple Orthogonal State Contrast (OSC) technique^2^, to the most complete and complex Mueller polarimetric imaging^11^ technique, enabling the comprehensive polarimetric characterization of a sample. In particular, Mueller polarimetric imaging shows promise to significantly improve the accuracy of screening for cervical cancer^12–16^, which continues to be the second most common type of cancer for females worldwide. Preliminary results obtained *ex vivo* on fresh cervical specimens showed that healthy tissues are characterized by a strong anisotropy^12^ which disappears in presence of pre-cancerous lesions. This effect has been quantitatively evaluated in a recent study where Mueller polarimetric imaging has been used to analyze seventeen samples of fixed cervical specimens^14^. The quantification of tissue anisotropy modification enables one to differentiate between high-grade cervical dysplasia and healthy squamous epithelium with an averaged value of both sensitivity and specificity of about 80% at a wavelength of 550 nm. The intense anisotropy observed in healthy squamous epithelium was interpreted as the signature of the structured and ordered collagen that forms the connective tissue beneath the healthy epithelium itself^13^. A pre-cancerous evolution in the epithelium can degrade the structure of said collagen fibers in the nearby connective tissue^17^, with the consequence of decrease in its macroscopically measurable anisotropy. Light scattering also decreases close to dysplatic epithelium. This effect is attributed to the decrease of the volume of collagen and the degradation of its structure^18^.

In current screening practice, colposcopy, consisting of the examination of the cervix and vagina by means of a low magnification microscope (named a colposcope) after successive application of acetic acid and iodine, only enables one to evaluate the difference in the scattering properties of epithelial nuclei for healthy and pathological regions. Practitioners can also use green light, which is strongly absorbed by hemoglobin, to highlight the presence of atypical blood vessels in the connective tissue that can be the signature of the presence of a malignant lesion in the epithelium. However, taken alone, colposcopy is strongly operator-dependent, with only 60–70% sensitivity and 50% specificity for high-grade cervical dysplasia detection, and with only a moderate inter-operator reproducibility (*κ* = 0.35)^19–25^. The *in vivo* detection and quantification of modifications in connective tissue induced by a premalignant lesion can be crucial to substantially improve cervical cancer screening. Previous *ex vivo* results show that Mueller polarimetric imaging is a good candidate to fill this purpose. However, the measurement of Mueller matrix images *in vivo* poses an instrumental challenge: the device should be compact, it should capture accurate images in real-world conditions “in the field” and it should be capable of performing the rapid acquisition of all the 16 images necessary to compute the Mueller Matrix. Hence, the *in vivo* polarimetric systems presented to date are almost exclusively based on a relatively simple technique using Orthogonal State Contrast (OSC)^2, 26^ polarimetry. Several types of endoscopic polarimetric systems (needed for the exploration of inner cavities of the human body) have also been proposed in recent years^27–32^, but they have only been tested on *ex vivo* tissue samples. Very few *in vivo* Mueller polarimetric systems have been demonstrated and/or tested, such as one for studying the collagenase of mice^33^, and another for application in opthalmology for human patients^34^.

In the present study, the first Mueller Polarimetric Colposcope (MPC) for *in vivo* analysis of the uterine cervix is presented. For this purpose, a miniaturized Mueller polarimetric imager has been grafted onto a classic colposcope. This new imaging tool enables the fast acquisition of Mueller polarimetric images, thus eliminating blur effects due to patient movement. This system has been tested for ease of use by a practitioner by analyzing *in vivo* the uterine cervix of a number of patients. The MPC was used in real-world conditions for a feasibility study. For this reason, only healthy uterine cervices of women with normal Pap smear were analyzed.

The paper is structured as follows: in the first section the MPC design is described; in the second part, *ex vivo* tests to ensure the reliability of MPC measurements are shown. In the third part, we show the results of *ex vivo* measurements that enabled us to determine conditions giving a measurement time suitable for *in vivo* applications. Finally, *in vivo* Mueller polarimetric measurements performed in real-world conditions on two patients are shown and discussed. The calibration method of the MPC and the interpretation of measured Mueller matrices are described in the “Methods” section. The “Discussion” section summarizes the conclusions and outlines perspectives for further developments.

## Results

### Mueller polarimetric colposcope design

**Figure 1:**
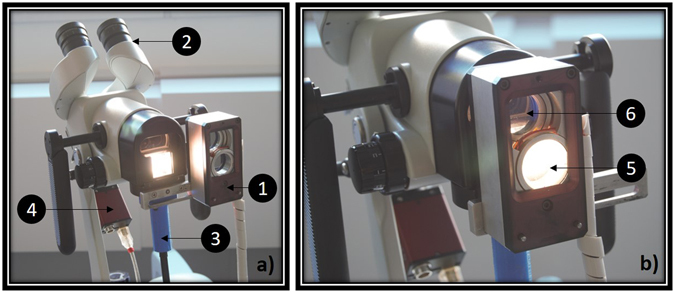
(**a**) Photo of the entire MPC system (1: Polarimetric head, 2: Binoculars for stereoscopic vision of the uterine cervix, 3: Liquid light guide, 4: CCD camera). The polarimetric head is in the “OFF” position, and permit classical colposcopic observations. (**b**) Polarimetric system can be slid on the “ON” position to permit the acquisition of Mueller polarimetric images (5: Polarization States Generator (PSG), 6: Polarization States Analyzer (PSA)).

A colposcope is a stereoscopic binocular microscope which enables the illumination and observation of the uterine cervix under low magnification (between 4× and 6×) ^35^. The colposcope used in this study, shown in Fig. 1(a), is an Olympus OSC 500 and is one of the most commonly used for colposcopy. The light is delivered by a 150 W halogen lamp source (Olympus CLH-SC), and is usually guided by a silica fiber bundle to the colposcope. In order to increase the amount of light incident on the uterine cervix under analysis, a liquid light guide (Thorlabs LLG0538-6) has been used instead of the fiber bundle due to its better transmission performances in the visible spectral range (340 nm–800 nm). In the classical colposcope design, a lens collimates the light exiting the flexible light guide. The position of the end of the light guide has been optimized and placed near the focal plane of this lens in order to reduce the spot size of the beam from 15 cm to 6 cm at working distance of 30 cm. This working distance is the typically mean distance between the colposcope and the uterine cervix under real-world conditions.

In order to perform measurements of the Mueller matrix images, a miniaturized Mueller polarimeter has been constructed and placed in front of the colposcope. As can be seen in Fig. 1, this Mueller polarimeter is framed within an airtight metallic box which measures 30 × 50 × 100 mm^3^ and is protected against dust and blood projections. As can be seen in Fig. 1(a), it can be slid either on the “OFF” position, in order to permit classical colposcopic observations, or on the “ON” position to perform the polarimetric measurements (Fig. 1(b)).

A Mueller matrix **M** is a 4 × 4 matrix with real coefficients which represents the signature of the complete polarimetric response of a sample. This matrix operates on a 4 × 1 Stokes vector (which represents an incident polarization state falling on the sample) to create a new transformed 4 × 1 Stokes vector (which represents a backscattered polarization state coming from the sample). Therefore, the determination of a Mueller matrix requires sixteen intensity measurements involving all possible incident and measured polarization states.

In the “ON” position, incoming light exiting the liquid light guide passes through the PSG (Polarization States Generator) of the Mueller polarimeter. This PSG temporally modulates the incoming light polarization by generating four independent probing polarization states. These states are described by four Stokes vectors, which are grouped together as the columns of a modulation matrix called **W**. Backscattered light coming from the uterine cervix passes through the PSA (Polarization States Analyzer) placed in front of one of the two input channels of the binocular system. At the end of this channel, a monochromatic camera is used for the acquisition of the image. After they have interacted with the uterine cervix, each polarization state of light generated by the PSG is analyzed through four independent configurations of the PSA. These configurations are described by four analysis Stokes vectors, which are grouped as the rows of an analysis matrix called **A**. Sixteen intensity measurements can thus be performed behind the PSA, and are stacked into a matrix named **B**. The sixteen intensity measurements contained in the **B** matrix are achieved with a CCD camera (Allied Prosilica GT1920) at 550 nm using a bandpass dichroic filter (Thorlabs FB550-40, 40 nm FWHM) which has been placed in front of the camera. This system provides images with a size of 600 × 800 pixels, and is shown in Fig. 1(a).

Knowing both the **W** and **A** matrices (which can be determined through the calibration procedure described in the “Methods” section), the Mueller matrix **M** of the sample can be computed from the **B** matrix for each pixel. Previous studies showed that the relevant polarimetric parameters for analyzing the uterine cervix microstructure were the Depolarization (Δ) and Retardance (R)^12, 14^. Depolarization enables one to evaluate the scattering properties of tissue while Retardance to quantify its anisotropy, generally due to the presence of collagen fibers, which are known to be strongly birefringent. Finally, the orientation of the retardance eigenaxis, named Azimuth (*α*) in this paper, is a crucial parameter to determine the orientation of the collagen fibers^12^. All these polarimetric parameters can be extracted from **M** with an appropriate matrix decomposition, as explained in the “Methods” section.

### *Ex vivo* results

#### Comparison with reference measurements

**Figure 2:**
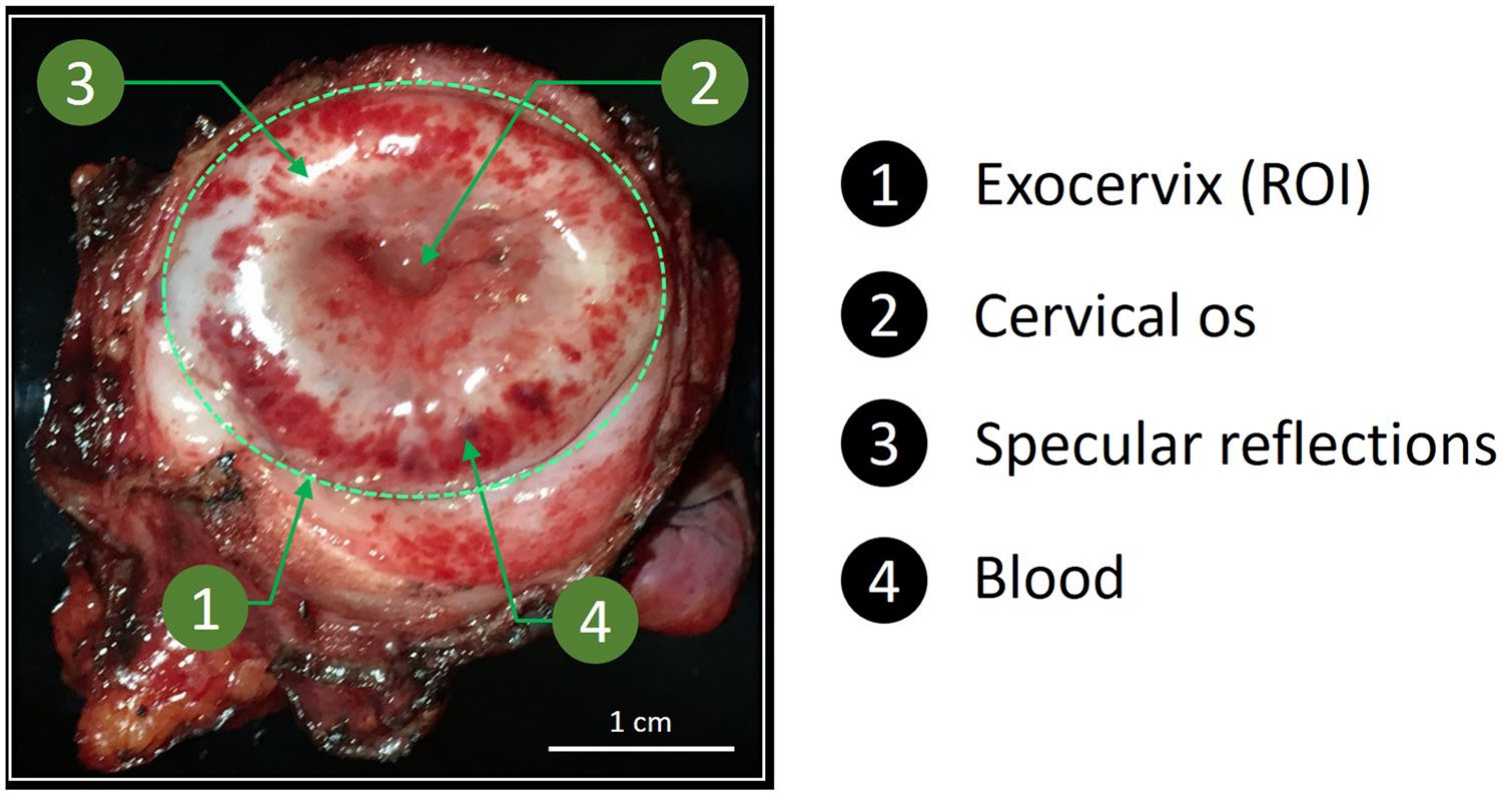
Unpolarized RGB picture of the uterine cervix from a complete hysterectomy from a patient with normal Pap smear.

The comparison described in this section has been performed in the Department of Gynecologic Pathology at the Cancer Center Gustave Roussy in Villejuif. The MPC has been used to analyze several specimens of *ex vivo* cervices from total hysterectomies of women having shown a normal Pap smear. It should be important to note that the measurements were performed on fresh tissues prior to any kind of fixations. These samples were then also analyzed using another system, a Mueller Imaging Polarimeter (MIP) in backscattering configuration described in previous studies^11, 12^ and routinely used for polarimetric imaging of *ex vivo* uterine cervix specimens. The MIP data are taken as reference measurements to determine the reliability of measurements obtained by the MPC. All analyzed samples have been delicately cleaned with fresh water after surgery and before performing any polarimetric image measurement. A typical example of analyzed specimen is shown in Fig. 2. Red spots present on the surface of the sample are thrombotic areas due to blood clots under the epithelium caused by surgery. White spots on the color picture correspond to saturated pixels of the camera and are due to the specular reflection of light on the sample surface.

**Figure 3:**
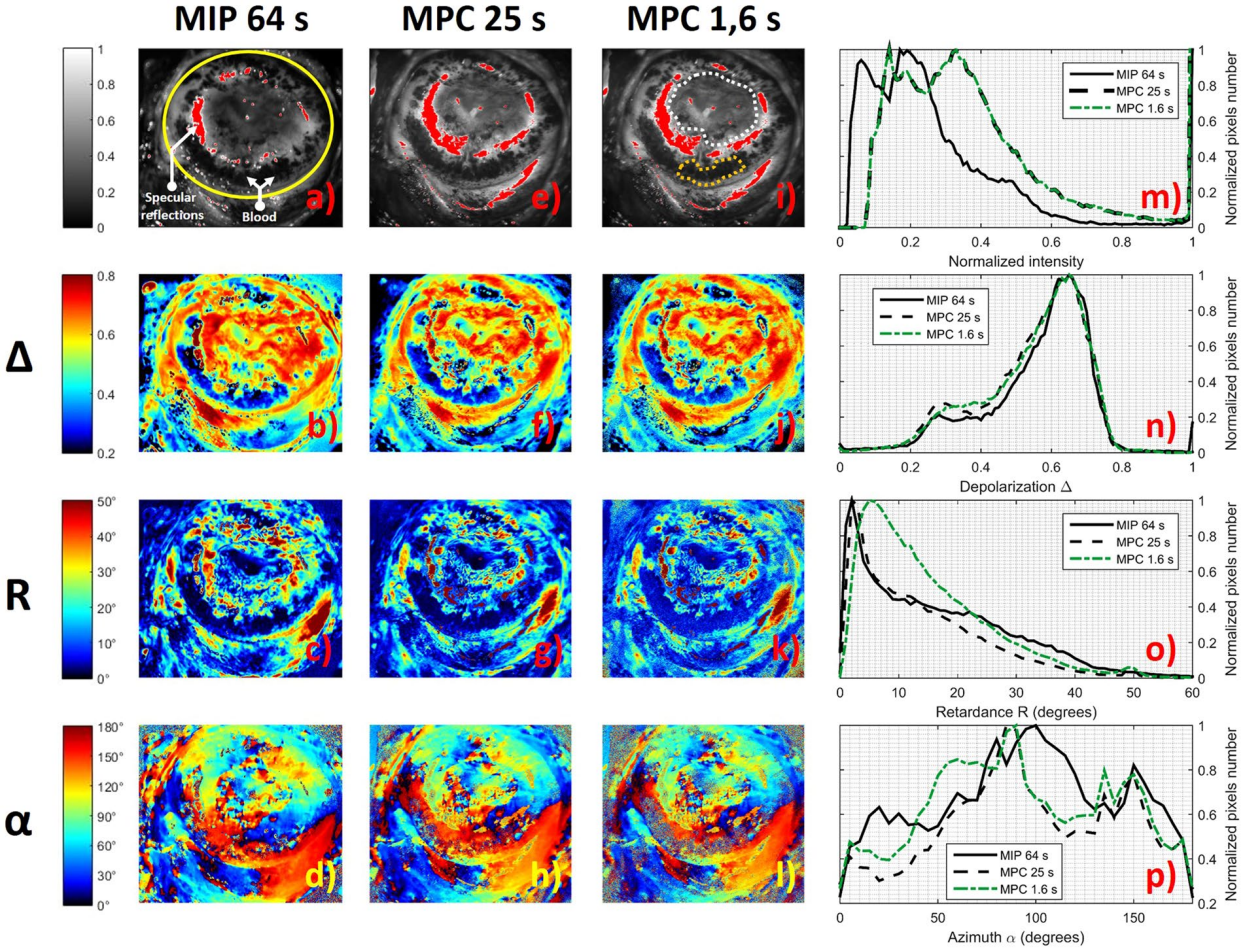
Comparison of intensity and polarimetric images obtained with the MIP and the MPC on the *ex vivo* uterine cervix shown in Fig. 2. (**a**–**d**) MIP Intensity image and polarimetric images of Depolarization (Δ), Retardance (*R*) and Azimuth (*α*) respectively for an acquisition time of 64 s. (**e**–**h**) the same parameters acquired by the MPC for an acquisition time of 25 s. (**i**–**l**) the same parameters acquired by the MPC for an acquisition time of 1.6 s. (**m**–**p**) histograms of Intensity, Depolarization, Retardance and Azimuth images respectively for both instruments at different acquisition times. All the histograms were calculated inside the ROI delineated by the yellow line in Fig. 3(a). White and orange dotted lines in Fig. 3(i) delimit two different areas that will be analyzed more in detail in Fig. 4.

The exposure time has been optimized by maximizing the signal on most of the image while minimizing the number of saturated pixels due to specular reflection (red zones in Fig. 3(a,e)). The most intense image amongst the sixteen images composing the intensity matrix **B** has been selected for this optimization. The resulting exposure times are 100 ms/image for the MPC and 250 ms/image for the MIP. The gains of both the MPC and MIP cameras were set to unity. In order to increase the signal to noise ratio (SNR), the intensity matrix was averaged 16 times, which led to total acquisition times of 25 s and 64 s for MPC and MIP respectively. The intensity images in Fig. 3(a,e) have been normalized with respect to the maximum of both the MPC and MIP cameras dynamic ranges. Due to the very different optical configurations of the two instruments (different sources, different cameras and different optical paths), the amount of collected light is not the same for each instrument and this leads to different optimal exposure times for the MIP and the MPC cameras. This can also explain why the zones displaying specular reflections are not exactly the same for the intensity images obtained with the MPC and the MIP (Fig. 3(a,e)), as well as why the respective histograms are not identical when superimposed (continuous and dotted black lines in Fig. 3(m)).

For both devices, the most relevant polarimetric parameters (namely the Depolarization (Δ), Retardance (*R*) and Azimuth (*α*)) were extracted from measured Mueller matrices by using the polar decomposition technique as explained in detail in the “Methods” section. Polarimetric parameters obtained by using the MPC are very close to those obtained by using the MIP; the Depolarization (Fig. 3(b–f)), Retardance (Fig. 3(c–g)) and Azimuth (Fig. 3(d–h)) appear to be very similar, despite observable differences between the intensity images (Fig. 3(a,e)). Superimposing the histograms of polarimetric images obtained by using the MPC and the MIP underlines their similarity, as shown in Fig. 3(n–p) for Depolarization, Retardance and Azimuth respectively (continuous and dotted black lines). All the histograms have been calculated for the Region Of Interest (ROI) of the sample marked by the yellow line in Fig. 3(a) and corresponding to the exocervix. The small differences observed for the Azimuth, as determined using the MIP and the MPC, are due to the orientation of the sample being not exactly the same for the two measurements. Indeed, the sample was first analyzed with the MIP and then it was moved in order to be analyzed with the MPC. For this reason, the MIP and MPC images are not perfectly superimposable and a pixelwise analysis is not possible in this case.

The presence of blood significantly modifies the polarimetric response of analyzed tissues. Indeed, the light is strongly absorbed by the hemoglobin at 550 nm and backscattered light from blood-rich zones is characterized by a very low value of Depolarization as shown in Fig. 3(b,f) and Retardance as shown in Fig. 3(c,g). Notably, the Azimuth is the only parameter which is less influenced by the presence of blood, as shown in Fig. 3(d,h). Finally, saturated pixels due to specular reflections are characterized by non-physical Mueller matrices, and are discarded. Overall, the results shown in this section confirm the reliability of the MPC measurements when benchmarked against measurements taken using the MIP, and motivate one to try to use this system in more exacting conditions, approaching those found in the field.

#### Reproducing conditions equivalent to *in vivo* measurements

The measurements shown above provide idealized polarimetric images that can be used as a reference, as they are obtained under maximized signal to noise ratio conditions. They also provide assurance of the reliability of measurements obtained by using the MPC through benchmarking. However, the required time for the acquisition of these images is not compatible with *in vivo* applications. Indeed, such a long acquisition time for the MPC (25 s) can lead to motion blur effects on the polarimetric images which can be due for example to the breathing movements or the heartbeat of the patient. A measurement time compatible with *in vivo* applications is between 1 and 2 s. To achieve such a measurement time, only a single acquisition is performed, while setting the camera parameters to an exposure time of 100 ms and a gain of unity. Under these conditions, the total time required to measure the full intensity matrix **B** is about 1.6 s. This value is reasonably well suited to eliminate any artefacts due to patient movements during the acquisition of the sixteen intensity images. The results obtained in 1.6 s appear visually similar to the reference images obtained in 25 s as shown in Fig. 3(e,i) for the Intensity, in Fig. 3(f,j) for Depolarization, in Fig. 3(g,k) for Retardance, Fig. 3(h,l) for Azimuth. The histograms showed in Fig. 3(m–p) were always calculated for the ROI marked by the yellow line in Fig. 3(a). Saturated pixels due to specular reflections are discarded because they are characterized by non-physical Mueller matrices. Histograms of intensity images are almost identical (green and black dotted lines in Fig. 3(m)). The similarity of the histograms for Depolarization is clearly seen by superimposing them (green and black dotted lines in Fig. 3(n)). However, more important differences are observed in the Retardance and Azimuth histograms obtained under the two measurement conditions. These differences are observed especially in thrombotic zones wherein light at a wavelength of 550 nm is strongly absorbed by the presence of blood, which leads to very low intensity signal strength.

**Figure 4:**
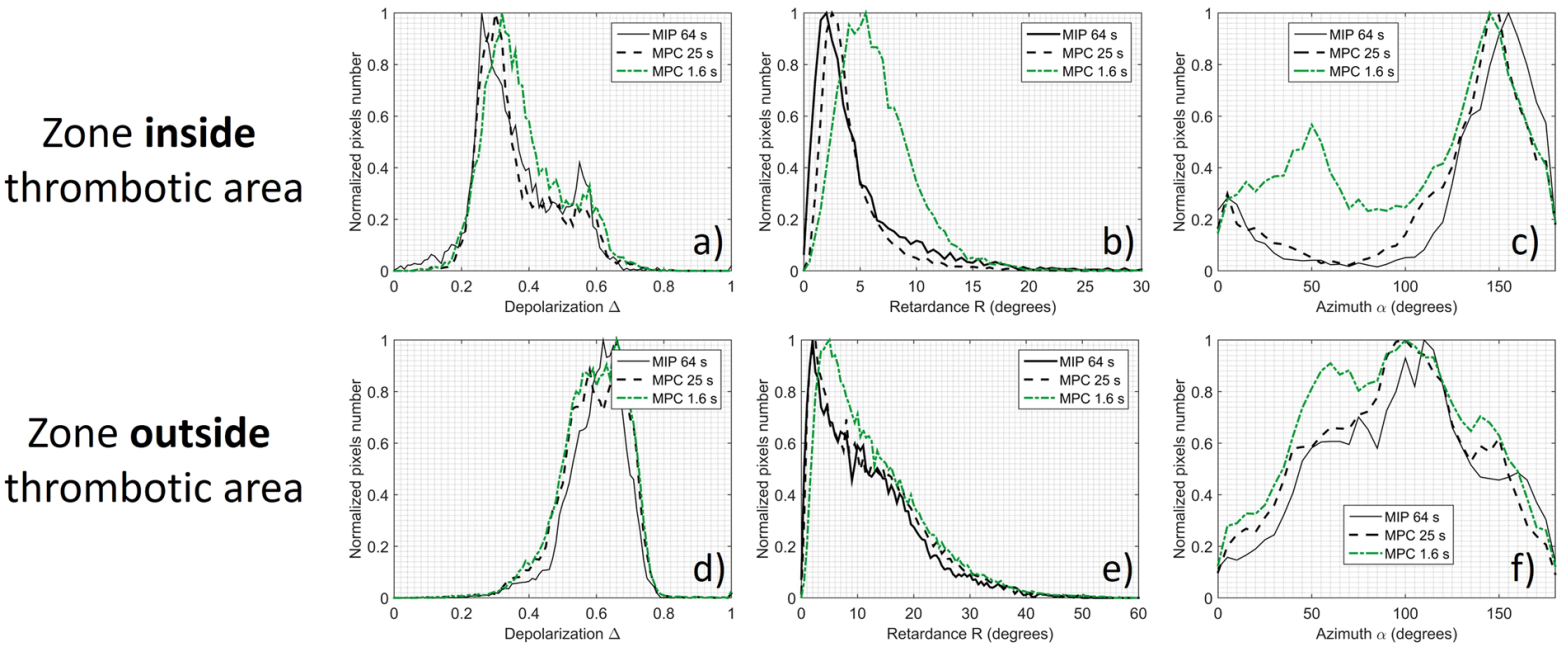
Comparison between histograms of Depolarization, Retardance and Azimuth measured with the MIP in 64 s (reference), with the MPC in 25 s and with the MPC in 1.6 s for the zone selected around the thrombotic zone (delineated by the orange dotted line in Fig. 3(i)) and the zone selected outside the thrombotic region (delineated by the white dotted line in Fig. 3(i)).

In order to show more clearly the effect of blood on polarimetric images, a zone around the thrombotic region (delineated by the orange dotted line in Fig. 3(i)) was selected and analyzed in more detail. For comparison, a second zone outside the thrombotic region (delineated by the white dotted line in Fig. 3(i)) was also selected and analyzed. For both selected zones, histograms of polarimetric parameters (Depolarization, Retardance and Azimuth) are compared in Fig. 4 for the MIP 64 s, MPC 25 s, and MPC 1.6 s measurements. As can be seen on this figure, the MIP 64 s and MPC 25 s measurements are very close to each other. Superimposing the histograms of polarimetric images acquired with the MPC in 25 s with those acquired in 1.6 s for the thrombotic region underlines their differences especially for Retardance and Azimuth. Values of Retardance obtained in 1.6 s are evidently larger than those obtained in 25 s (Fig. 4(b)). The Azimuth appears oriented around a peak value of 150° for the acquisition time of 25 s, while its value is more evenly distributed between 0° and 180° for the acquisition time of 1.6 s as shown in Fig. 4(c). These differences are less evident for Depolarization as shown in Fig. 4(a). The histograms of polarimetric images acquired with the MPC in 25 s and 1.6 s in the zone selected outside the thrombotic region are strictly similar for all polarimetric parameters as shown in Fig. 4(d–f) for Depolarization, Retardance and Azimuth respectively.

For the selected thrombotic zone, the values of the Azimuth and Retardance obtained with the MPC in 1.6 s are very different from those obtained in 25 s and from MIP reference measurements. It is clear that this effect is due to the strong absorption of blood at 550 nm which strongly decreases the signal to noise ratio. In this case, averaging several acquisitions of intensity matrices can greatly improve the quality of polarimetric images, but at the expense of acquisition time.

However, thrombotic areas observed *ex vivo* are essentially due to surgery and are generally absent *in vivo*. For this reason, the conditions found to perform measurements in 1.6 s are well suited for *in vivo* applications as it will be shown in the following.

### *In vivo* results

**Figure 5:**
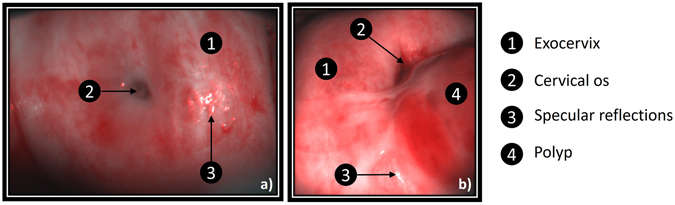
*In vivo* unpolarized RGB pictures of two uterine cervices. (**a**) Healthy uterine cervix (Cervix 1). (**b**) Healthy uterine cervix with polyp (Cervix 2).

Knowledge obtained from the *ex vivo* results shown above have both ensured the reliability of the MPC measurements and enabled one to determine the suitable experimental conditions for *in vivo* applications. Applying this knowlegde, *in vivo* measurements of uterine cervices were performed on a number of patients in the Gynecological Surgery Department at the Kremlin Bicêtre University Hospital in Paris. In this section, *in vivo* measurements are presented for two uterine cervices. The two measurements were taken before surgery while the two patients were under anesthesia. The first patient was a 53 years old woman hospitalized for a uterine fibroid. She underwent a total hysterectomy by laparotomy. The second patient was a 47 years old woman hospitalized for an endometrial ablation by hysteroscopy. Both analyzed uterine cervices shown in Fig. 5(a,b) are completely healthy: the Pap smear was normal for both patients and no malignancies were present on the explored cervices. However, the uterine cervix shown in Fig. 5(b) is characterized by the presence of an endocervical polyp visible to the naked eye and easily identified by the practitioner as a benign growth of cervical tissue.

White spots on the color pictures shown in Fig. 5 correspond to saturated camera pixels due to specular reflection of light off of the surface of the sample.

**Figure 6:**
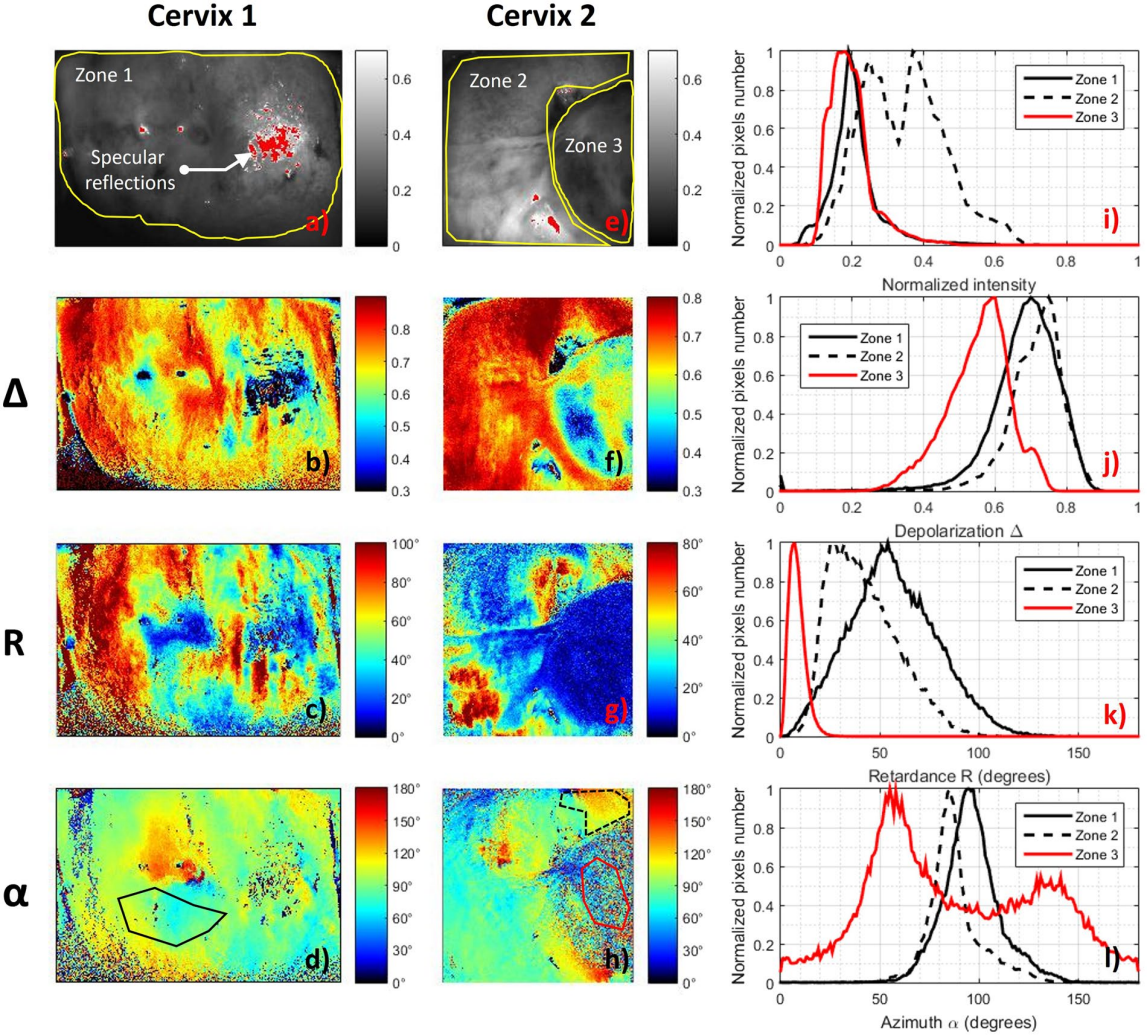
Comparison between intensity and polarimetric images of the *in vivo* uterine cervices shown in Fig. 5. (**a**–**d)** MPC intensity image and polarimetric images of Depolarization (Δ), Retardance (*R*) and Azimuth (*α*) respectively, for Cervix 1; (**e**–**h**) the same parameters for Cervix 2. Corresponding histograms of Intensity, Depolarization, Retardance and Azimuth images for both samples are plotted in the Fig. 6(i–l) respectively. Histograms were calculated inside the three ROI delineated by the yellow solid lines in Fig. 6(a,e) (Zone 1, Zone 2 and Zone 3). Red line in Fig. 6(h), solid and dotted black lines in Fig. 6(d,h) delineate three areas that will be analyzed more in detail in Fig. 7.

For the first patient, the selected ROI indicated as “Zone 1” in Fig. 6(a), corresponds to the exocervix. For the second patient, the first selected ROI, indicated as “Zone 2” in Fig. 6(e), corresponds to the exocervix while the second selected ROI, indicated as “Zone 3” in Fig. 6(e) corresponds to the polyp. The zones are delineated by yellow solid lines.

The exocervix is the external visible part of the cervix entirely covered by a stratified squamous (also named malpighian) epithelium which is composed by flattened cells arranged in layers (for a total thickness of about 300 µm). The polyp is covered by a simple columnar (also named glandular) epithelium which is composed by a mono-layer of elongated and column-shaped cells. The glandular epithelium generally covers the walls of the endocervical canal and should be not directly visible to the colposcopy. However, it often comes out the endocervical os and becomes visible due to benign modifications (ectropion) of the cervix (during pregnancy or in the ovulatory phase for younger women), or to the formation of endocervical polyps as described herein. Each epithelium is in direct contact with the basement membrane, a thin fibrous layer which separates it from the underlying connective tissue mainly composed by collagenous and elastic fibers. Both types of epithelia can be found on the uterine cervix surface directly accessible to the colposcopic examination. Polarimetric characterization of squamous and glandular epithelia for healthy cervical tissue is the start point to detect precancerous lesions unambiguously.

In order to obtain the polarimetric characterization *in vivo* of both described uterine cervices with the MPC, a single acquisition has been performed by setting the camera exposure time to 100 ms and the gain to unity as stated in the previous section, giving a total measurement time of 1.6 s. The MPC was used with ease by the surgeon, as though it was a classic colposcope under real-world conditions.

Healthy malpighian tissue is characterized by a strong anisotropy. Indeed, mean values of 60° and 40° for the Retardance have been measured for Zone 1 and Zone 2 respectively as shown in Fig. 6(c,g). A mean value of about 10° has been measured for Zone 3 as shown in Fig. 6(g). Retardance can be a relevant polarimetric parameter to distinguish malpighian from glandular epithelium. Indeed, when superimposing the histograms for the polyp and for the malpighian epithelium, little overlap is observed, as shown in Fig. 6(k). The polyp also appears to be less depolarizing than the healthy tissue. A mean value of 0.75 has been measured for Zone 1 and Zone 2 as shown in Fig. 6(b–f) while the mean value of the Depolarization in Zone 3 is about 0.6, as shown in Fig. 6(f). An overlap between the histograms is observed due to the spatial inhomogeneity of this parameter within the malpighian epithelium, in particular for Cervix 1. The spatial variation of Retardance and Depolarization are due to the presence of specular reflections in certain zones and to spatial inhomogeneity of vascularization which can strongly affect the determination of these parameters. Finally, the Azimuth appears to be oriented around 90° for both Zone 1 and Zone 2 as shown in Fig. 6(d–h) while it is characterized by a pixelwise inhomogeneity in the Zone 3 as shown in Fig. 6(h). The strong Retardance observed in the zones around the malpighian epithelium has been attributed to the signature of a well-ordered collagen layer composing the subepithelial connective tissue^13^. Otherwise, the polyp does not show a well-defined inner organization in the subepthelial connective tissue, which results in low Retardance values as well as erratic Azimuth orientations, as can be seen in Fig. 6(g,h). In particular, the value of the Azimuth varies between 0° and 180° with strong pixel to pixel variation (Fig. 6(l)).

**Figure 7:**
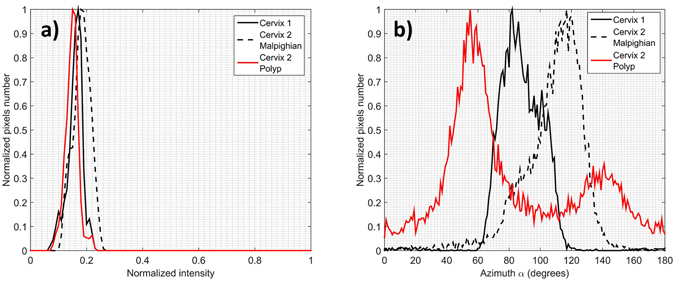
Histograms of Intensity (**a**) and Azimuth (**b**) of areas delineated in Fig. 6(d,h).

In order to verify that the pixelwise variation of the Azimuth observed on the polyp does not result from low signal strength (such it is the case for thrombotic zones previously discussed for *ex vivo* measurements), a zone around the polyp region (marked by the red solid line in Fig. 6(h)) was selected and analyzed in more detail. For comparison, two other zones were selected around the malpighian epithelium of the two analyzed uterine cervices (solid and dotted black line in Fig. 6(d,h)). The three selected areas are characterized by the same amount of collected light intensity as shown in Fig. 7(a). The Azimuth measured in malpighian tissue is characterized by a well-defined direction (around of 80° for the Cervix 1 and around of 120° for the Cervix 2) while it is widely distributed between 0° and 180° for the polyp, as can be seen on the Fig. 7(b). This result clearly shows that the distributed orientations of the Azimuth on the polyp it is not due to a lack of light but to the different microscopic structure of the polyp.

**Figure 8:**
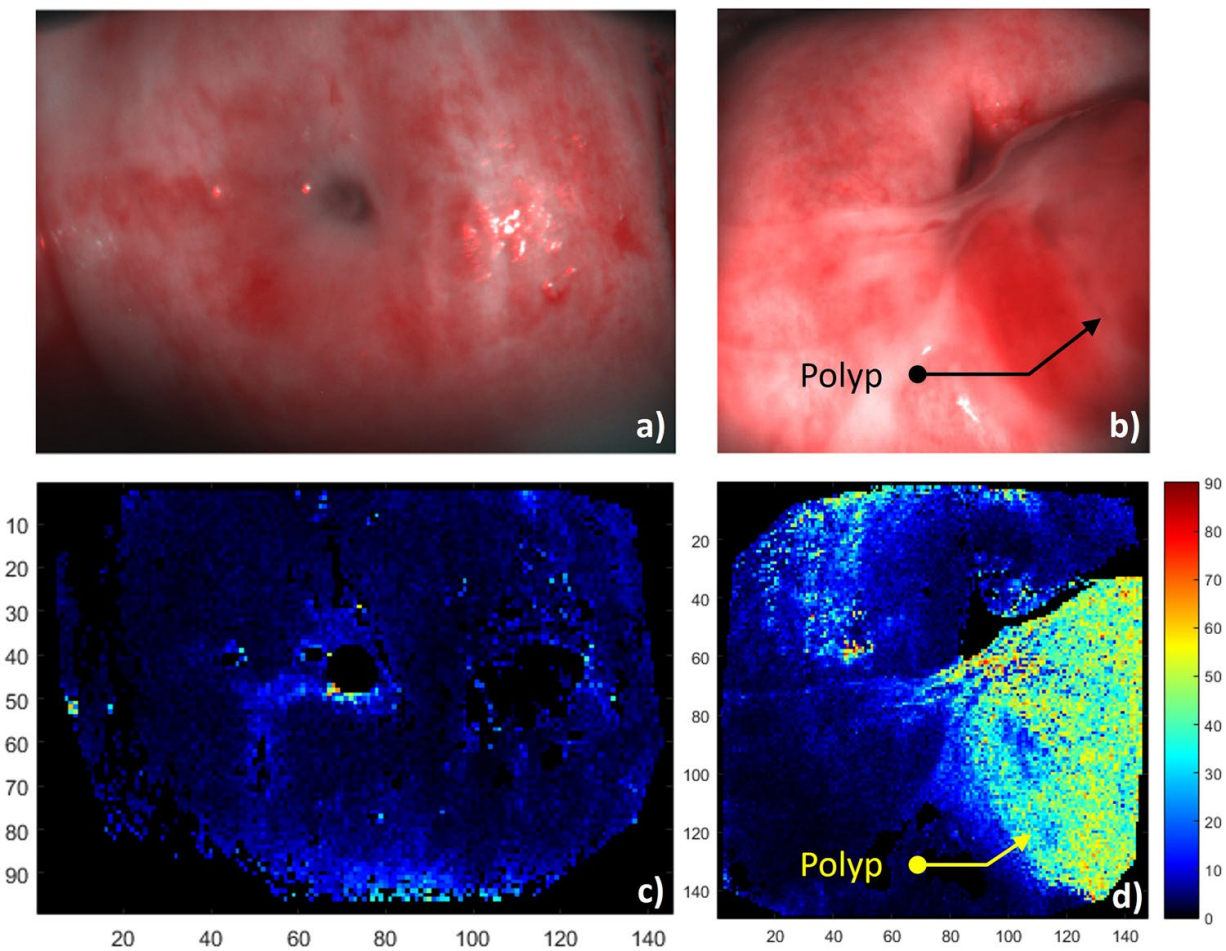
(**a**,**b**) Unpolarized RGB pictures of Cervix 1 and Cervix 2 respectively. (**c**,**d**) Standard deviation of the Azimuth computed for each pack of 3 × 3 pixels.

The pixelwise variation of the Azimuth orientation in disorganized areas translates into a change in the texture of the Azimuth image. This can be used to create a contrast based on the spatial variation in the organization of the tissue. To do so, the standard deviation *σ*(*α*) of the Azimuth has been calculated for each pack of 3 × 3 pixels and is shown in Fig. 8. Healthy tissue exhibits weak values of *σ*(*α*): a mean value of 7.2° is obtained on Zone 1 of Cervix 1 and 6.7° on Zone 2 of Cervix 2. Contrarily, the polyp region has a much larger value of 37.2°, due to the high variability of the Azimuth between each pixel. Pixels corresponding to very low intensity levels (less than 5% of the dynamic range of the camera) have been eliminated from this computation. These pixels are essentially located at the boundaries of the images and on the endocervical os. Pixels corresponding to saturated intensities have also been eliminated. This method allows one to significantly enhance the visual contrast between the polyp and the healthy areas and then to clearly separate the two types of tissues (Fig. 8).

## Discussion

In this work, the first Mueller Polarimetric Colposcope (MPC) capable of the *in vivo* analysis of the uterine cervix has been presented. The MPC was fabricated with minimal modification of a classic colposcope, as is currently used for cervical cancer screening. This new imaging tool can be easily used by a practitioner without changing their current practices and enables the fast acquisition of reliable *in vivo* Mueller polarimetric images, thus eliminating any blurring effects due to patient movements.

The design, calibration and image analysis procedure have been specified above. The MPC has been tested previously on a number of *ex vivo* healthy cervical specimens from total hysterectomies of women displaying normal Pap smear results. These samples were also analyzed with a Mueller Imaging Polarimeter (MIP) in backscattering configuration, routinely used for polarimetric imaging of *ex vivo* cervical specimens. The MIP measurements were used as a reference to confirm the accuracy of the data obtained with the MPC. Then, still using the *ex vivo* cervical specimens, a set of suitable operating conditions were determined for the MPC to achieve an acquisition time compatible with *in vivo* measurements. Finally the MPC was tested *in vivo* in real-world conditions on a number of healthy uterine cervices for patients with normal Pap smear.

We have shown the ability of Mueller polarimetric imaging to clearly distinguish the two main types of epithelia (malpighian and glandular) that can be found on a healthy uterine cervix. Our results *in vivo* confirmed that the malpighian tissue is characterized by a strong anisotropy and so by high Retardance values as widely showed in previous studies *ex vivo*. On the opposite, we observed that glandular epithelium is characterized by a very low Retardance. This observation can introduce a difficulty in the detection of precancerous lesions which are also characterized by a weak anisotropy^14^. Future work will be dedicated to make possible an unambiguous distinction between a precancerous lesion and glandular epithelium by using, for example, a multi-spectral approach.

## Methods

### Structure of the Mueller polarimeter and Mueller matrix determination

The polarimetric head shown in Fig. 1 contains both a PSG (Polarization States Generator) and a PSA (Polarization States Analyzer). The PSG consists of an assembly containing a linear polarizer followed by two tunable ferroelectric liquid crystal cells, sandwiching a quarter waveplate. The PSA is composed by the same elements as the PSG, but in the reverse order. As explained in the first part of the “Results” section, sixteen intensity measurements are performed and stacked into a matrix **B** for each pixel of the camera. This matrix **B** can be expressed as:

1 $${\bf{B}}={\bf{AMW}}$$

where **M** is the Mueller matrix of the uterine cervix. **W** and **A** can be retrieved through the calibration procedure explained in the next section. With their knowledge, one can easily find **M** by computing:

2 $${\bf{M}}={{\bf{A}}}^{-{\bf{1}}}{\bf{B}}{{\bf{W}}}^{-{\bf{1}}}$$

Obviously, equation (2) implies that both **W** and **A** are first non-singular matrices, and are well conditioned to minimize the error propagation between **B** and **M**. This was achieved by finding orientations of the liquid crystal cells’ neutral axes which minimize the condition numbers of both **W** and **A** as much as possible. More detailed information about the design and the optimization of this sequential Mueller polarimeter can be found in ref. 36. Synchronization of the voltages applied on the ferroelectric liquid crystal cells of the Mueller polarimeter with data collection from the camera is performed under LabVIEW.

### Calibration

Calibrations of both the MPC and the MIP were performed by the Eigenvalues Calibration Method (ECM)^37^, which provides accurate measurements of the Mueller matrices at 550 nm. This method avoids tedious polarimetric characterization and modelization of optical components in the PSG and PSA because it intrinsically takes into account all the polarimetric imperfections which can be present in the optical path such as residual diattenuation and/or retardance, or spectral depolarization induced by the non-zero spectral bandwidth of the dichroic filter.

The ECM is used to retrieve **W** and **A** matrices for each pixel. For this purpose, the ECM necessitates making a set of independent intensity measurements on a number of calibrated samples placed in the optical path and whose polarimetric characteristics are perfectly known. These calibrated samples must be well chosen in order to get the best estimate of both **W** and **A**. Previous studies have shown that a convenient set of calibrated samples were found to be a polarizer at 0°, a polarizer at 90°, and a waveplate providing a linear retardance between 80° and 140°, with one of its neutral axis set at 30°^38^. Accordingly, the following calibrated samples were placed one after the other in front of the PSG:

1. a first polarizer (Melles Griot FPG003) set at 0° (vertical) then at 90°, which are respectively called “P0” and “P90”;
2. a true zero order quarter waveplate (CASIX WPF 1225 *λ*/4–532) with a 90° linear retardance at 532 nm named “L30”.

As stated in the previous section, the MPC is used in backscattering configuration and should therefore calibrated in the same condition. For this purpose, a sandblasted metallic plate has been placed at approximately 30 cm in front of the colposcope to reflect the light passing through the PSG and the calibrated samples towards the PSA. The same sandblasted metallic plate was used on the MIP to make the measurements in backscattering configuration. It is worth mentioning here that the ECM imposes that the Mueller matrix of this sandblasted metallic plate alone, when used as a reflector, must be the identity matrix^37^.

The total amount of time needed to calibrate the MPC or the MIP for one wavelength is less than two minutes, including intensity measurements made on calibrated samples. The calculation of **W** and **A** matrices has been performed using Matlab. Images of these modulation and analysis matrices have been placed in the “Supplementary Information” file attached to this article. After the calibration process was completed, the orientations of the P0, P90 and L30 computed by ECM algorithm were respectively equal to 0°, 91.1° and 30.2° for the MPC, and respectively equal to 0°, 90° and 32.9° for the MIP. The small differences between these experimental values and the expected ones can be due to slight orientational misalignments of the calibrated components relative to each other during the calibration procedure.

Then, intensity matrices **B** of calibrated components P0, P90, L30 have been measured again, and their corresponding Mueller matrices have been computed by applying the equation (2) on each pixel. For clarity purposes, all of the Mueller matrix images of these components along with their histograms have been placed in the “Supplementary Information” file. Maximum discrepancies between the theoretical mean values of these histograms and the experimental ones were found to be lower than 0.017 for the MPC, and lower than 0.026 for the MIP on all Mueller matrix images.

### Mueller matrices interpretation

Once the Mueller matrix images have been measured, the sample’s polarimetric properties can be retrieved by a number of procedures. In this study, we made the choice to interpret these images by applying the polar decomposition proposed by Lu and Chipman in 1996^39^, which is the most widely used in tissue polarimetry. This decomposition describes a Mueller matrix **M** as a product of three matrices:

3 $${\bf{M}}={{\bf{M}}}_{{\rm{\Delta }}}{{\bf{M}}}_{R}{{\bf{M}}}_{D}$$

where **M**_Δ_, **M**_*R*_ and **M**_*D*_ are respectively the Mueller matrices of a depolarizer, a retarder, and a diattenuator from which the polarimetric properties can be extracted. Assuming that all the analyzed Mueller matrices are normalized by their **M**_11_ term before decomposition, diattenuation parameters such as linear diattenuation *D*_*L*_ and circular diattenuation *D*_*C*_ are given by:

4 $${D}_{L}=\sqrt{{{\bf{M}}}_{12}^{2}+{{\bf{M}}}_{13}^{2}}$$

5 $${D}_{C}=|{{\bf{M}}}_{14}|$$

The linear and circular diattenuation parameters express the sample’s capacity to unequally attenuate two orthogonal linear and circular polarization states, respectively. For its part, depolarization parameter Δ of a sample is given by:

6 $${\rm{\Delta }}=1-\frac{|a|+|b|+|c|}{3}$$

where a, b and c are the **M**_Δ_ eigenvalues. Let us note these eigenvalues bring information on the whether the sample depolarizes all the polarization states in the same manner, i.e. if a = b = c (isotropic depolarizer), or tends to depolarize linear and circular polarization states differently (a = b ≠ c). As a consequence, Δ reflects the total depolarization power of the Mueller matrix, and includes the sample’s ability to depolarize both linear and circular polarization states. Δ can range from 0 to 1 in the case of a non-depolarizing or a pure depolarizing sample respectively. At last, the retardance property *R* due to sample’s birefringence can be computed using the following formula:

7 $$R=\arccos (\frac{tr({{\bf{M}}}_{R})}{2}-1)$$

where *tr*(**M**_*R*_) denotes the trace of the **M**_*R*_ matrix. Retardance *R* represents the combined effect of linear retardance *R*_*L*_ and circular retardance *R*_*C*_. However, many studies revealed that linear retardance is largely dominant in biological tissues; indeed, polarimetric measurements performed on such tissues in backscattering configuration revealed that this type of structures exhibits very low circular retardance^1, 13, 40, 41^. Consequently, *R* ≈ *R*_*L*_, and the Mueller matrix **M**_*R*_ can be determined by^32, 40^:

8 $${{\bf{M}}}_{R}=[\begin{array}{cccc}1 & 0 & 0 & 0\\ 0 & {\cos }^{2}2\alpha +{\sin }^{2}2\alpha \,\cos \,R & \cos \,2\alpha \,\sin \,2\alpha (1-\cos R) & -\,\sin \,2\alpha \,\sin \,R\\ 0 & \cos \,2\alpha \,\sin \,2\alpha (1-\cos R) & {\sin }^{2}2\alpha +{\cos }^{2}2\alpha \,\cos \,R & \cos \,2\alpha \,\sin \,R\\ 0 & \sin \,2\alpha \,\sin \,R & -\,\cos \,2\alpha \,\sin \,R & \cos \,R\end{array}]$$

where *α* is the orientation of one eigenaxis of the sample (also named “Azimuth” in this article), and can be determined using the following formula:

9 $$\alpha =\frac{1}{2}\arctan (\frac{{{\bf{M}}}_{R24}}{{{\bf{M}}}_{R43}})$$

The use of the “arctan” function imposes *α* to vary within a range from [0°-90°]. In order to avoid recurring jumps on the colormaps and so to facilitate the reading of the *α* values on a whole polarimetric image, this range can be extended up to [0°-180°] by using the sign of the **M**_*R*24_ term if one supposes sin*R* > 0 (i.e. 0° < *R* < 180°). If this sign becomes positive, it means *α* has exceeded 90°: in this case, a 90° offset is added to the raw *α* value computed by the relation given in equation (9). The assumption of 0° < *R* < 180° is valid since no studies in tissue polarimetry, to our knowledge, have shown retardance values neither greater nor close to 180°. Retardance values found in this article on uterine cervices, known to be strongly birefringent organs, confirm this assumption.

Finally, it should be noted that although effective, the polar decomposition used in this paper may not be the best manner to interpret the measured Mueller images. Indeed, more recent procedures such as symmetric or logarithmic decompositions^42, 43^ could be more suitable to describe the polarimetric response of a biological tissue.

### Ethical statements concerning experiments on humans

The authors confirm that all experiments were performed in accordance with relevant guidelines and regulations. All experimental protocols and human work methods were carried out in accordance with procedures that were approved by the “Institut National du Cancer (INCa)” and the “Cancéropôle”. Informed consent was obtained from all subjects.

### Ethical rules

This work was funded by the “Institut National du Cancer (INCa)” and the “Cancéropôle”, under contract 2012-1-GYN-01-EP-1. The study was conducted under the supervision of the “INCa” and “Cancéropôle” that guaranteed that the internationally accepted principles and practices related to the ethical conduct of research involving the use of human subjects or animals were observed in this study.

## Electronic supplementary material

Supplementary Information
