# Supplementary material for: In vivo imaging of uterine cervix with a Mueller polarimetric colposcope
Source: Sci Rep. 2017 Jun 1;7:2471. doi: 10.1038/s41598-017-02645-9 (PMC5453972; doi:10.1038/s41598-017-02645-9)
Supplement: Supplementary file 1 — Supplementary Information [file 41598_2017_2645_MOESM1_ESM.pdf]

In vivo imaging of uterine cervix with a Mueller polarimetric colposcope

Supplementary information

Jérémy Vizet, Jean Rehbinder, Stanislas Deby, Stéphane Roussel, André Nazac, Ranya Soufan, Catherine Genestie, Christine Haie-Meder, Hervé Fernandez, François Moreau and Angelo Pierangelo

The aim of this document is to provide the Mueller matrices of calibrated samples measured by both the MPC (Mueller Polarimetric Colposcope) and the MIP (Mueller Imaging Polarimeter) at 550 nm. Modulation and analysis matrices **W** and **A** of the two instruments are also shown.

1 Calibration of the MPC

1.1 Mueller matrices of calibrated samples

Supplementary Fig. S1 shows the Mueller matrix of the sandblasted metallic plate, which is very close to the identity matrix. Mueller matrices obtained on P0, P90, and L30 components are also exhibited on Supplementary Fig. S3, Supplementary Fig. S5 and on Supplementary Fig. S7, respectively.

Corresponding histograms of these Mueller matrices are also displayed below on Supplementary Fig. S2, Supplementary Fig. S4, Supplementary Fig. S6, and on Supplementary Fig. S8, respectively. These were calculated in the Region of Interest (ROI) delineated by the dashed yellow line. This ROI corresponds to the area where the Mueller matrices of the uterine cervices have been computed. Mean and standard deviation values of all histograms have been precised by the letters "μ" and "σ", respectively. Maximum discrepancies between the theoretical red-colored mean values μ of these histograms, and the experimental ones in black were found to be lower than 0.017.

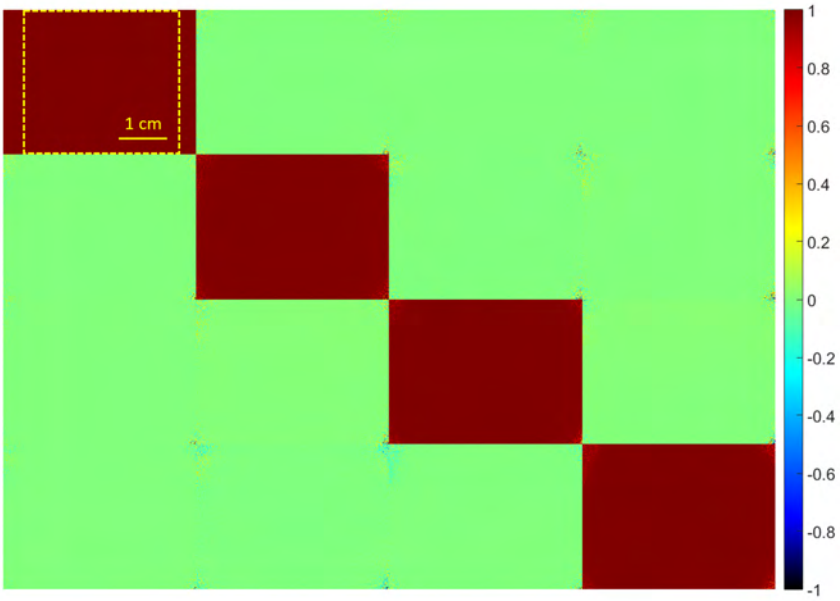

Supplementary Figure S 1: Mueller matrix of the metallic sandblasted plate.

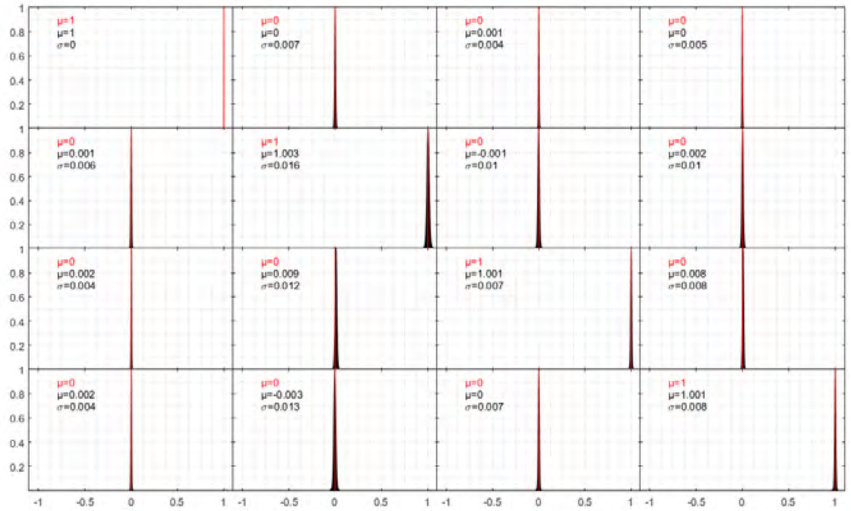

Supplementary Figure S 2: Histogram of the Mueller matrix displayed on Supplementary Fig. S1.

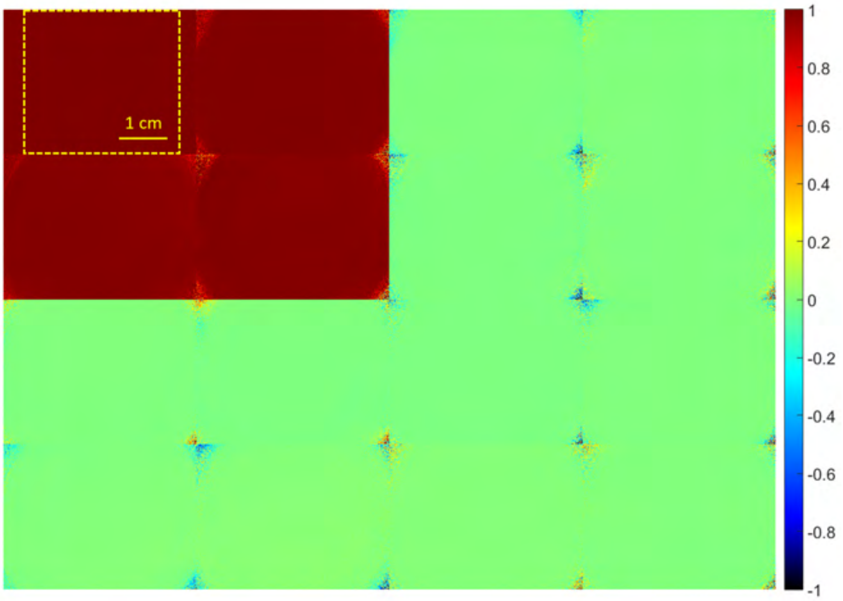

Supplementary Figure S 3: Mueller matrix of P0 component (linear polarizer at 0°).

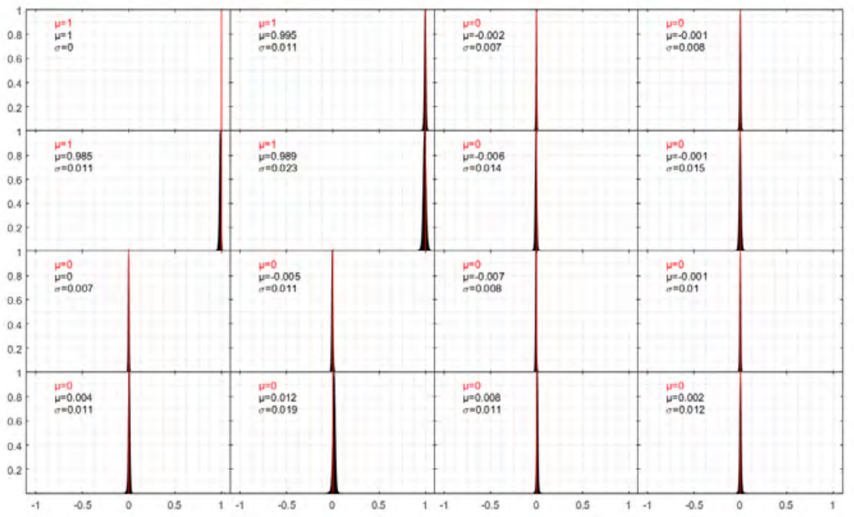

Supplementary Figure S 4: Histogram of the Mueller matrix displayed on Supplementary Fig. S3.

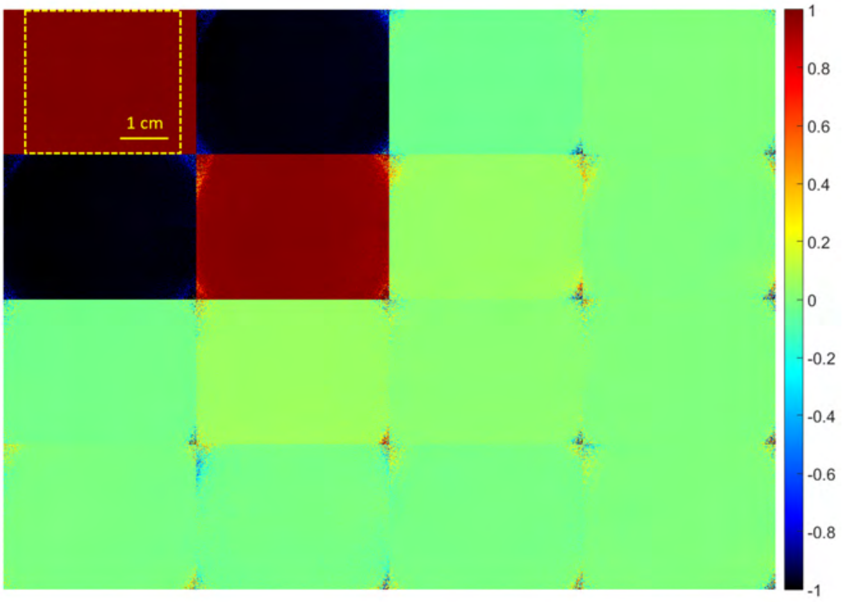

Supplementary Figure S 5: Mueller matrix of P90 component (linear polarizer at 91.1°).

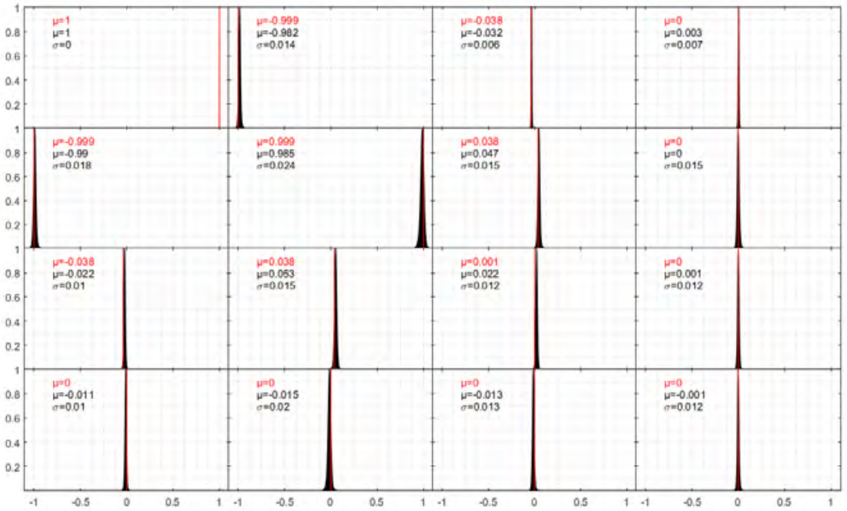

Supplementary Figure S 6: Histogram of the Mueller matrix displayed on Supplementary Fig. S5.

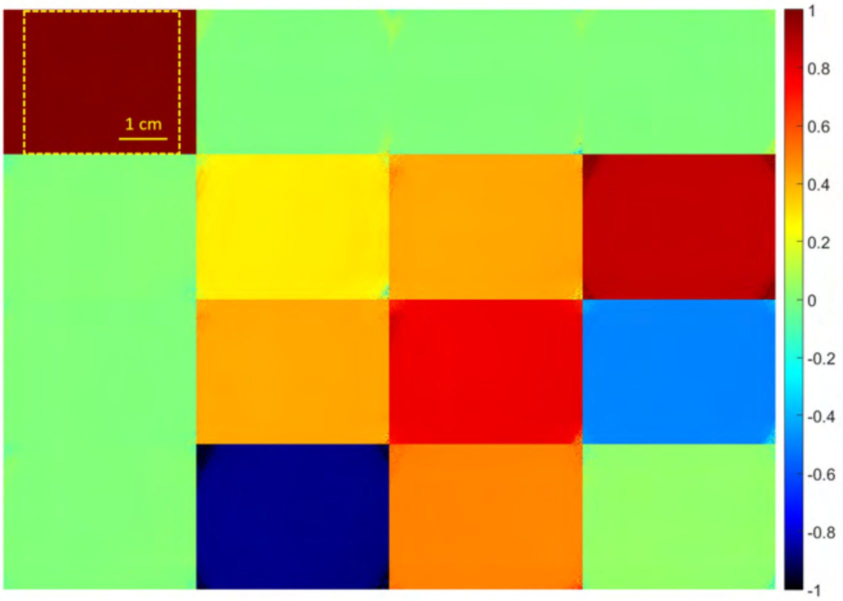

Supplementary Figure S 7: Mueller matrix of L30 component (quarter waveplate @ 532 nm at 30.3°).

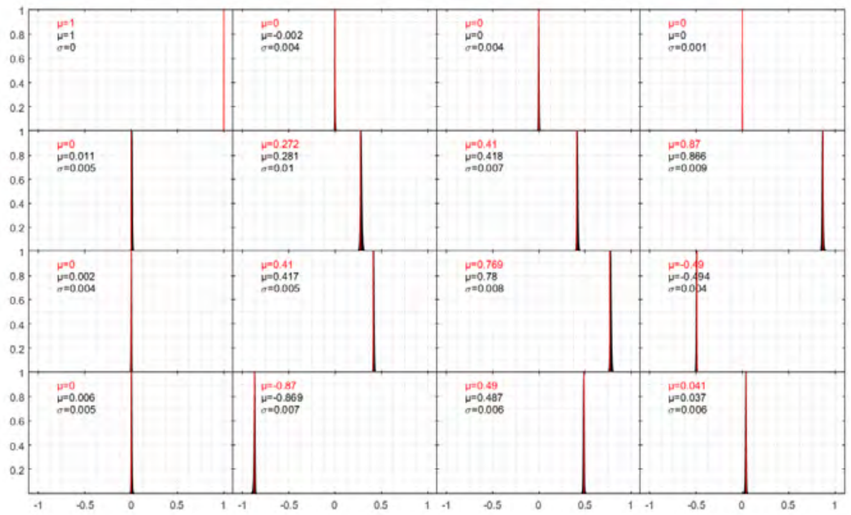

Supplementary Figure S 8: Histogram of the Mueller matrix displayed on Supplementary Fig. S7.

1.2 Modulation and analysis matrices

Supplementary Fig. S9 and Supplementary Fig. S11 show the modulation and analysis matrices **W** and **A** respectively. Corresponding histograms of these matrices are also displayed below on Supplementary Fig. S10 and Supplementary Fig. S12, respectively.

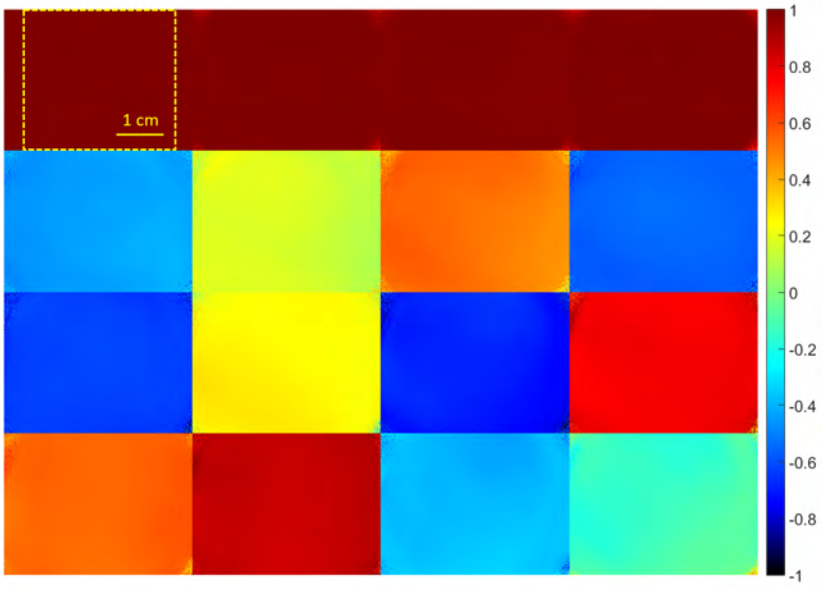

Supplementary Figure S 9: Modulation matrix **W**.

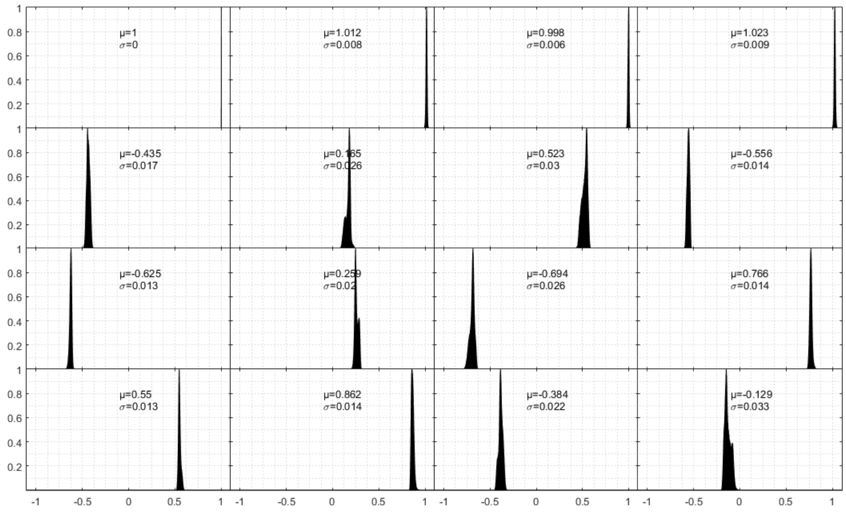

Supplementary Figure S 10: Histogram of the modulation matrix **W** displayed on Supplementary Fig. S9.

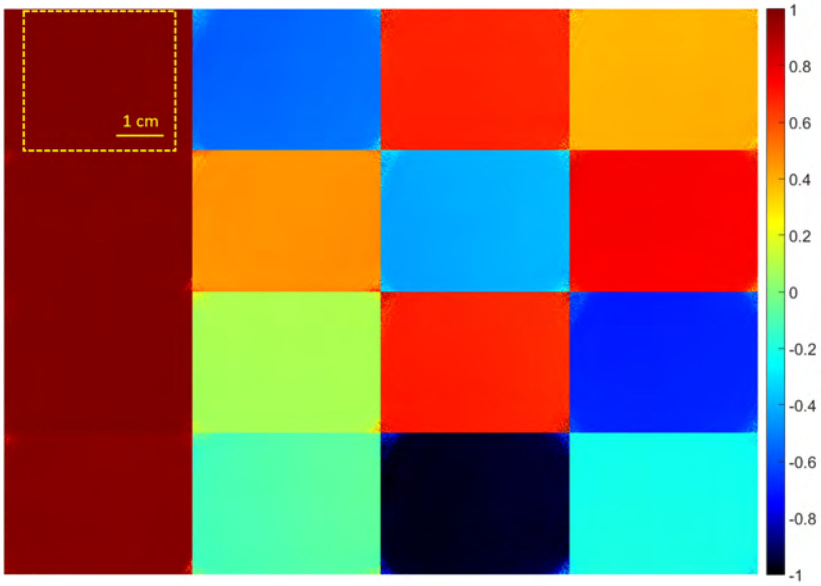

Supplementary Figure S 11: Analysis matrix **A**.

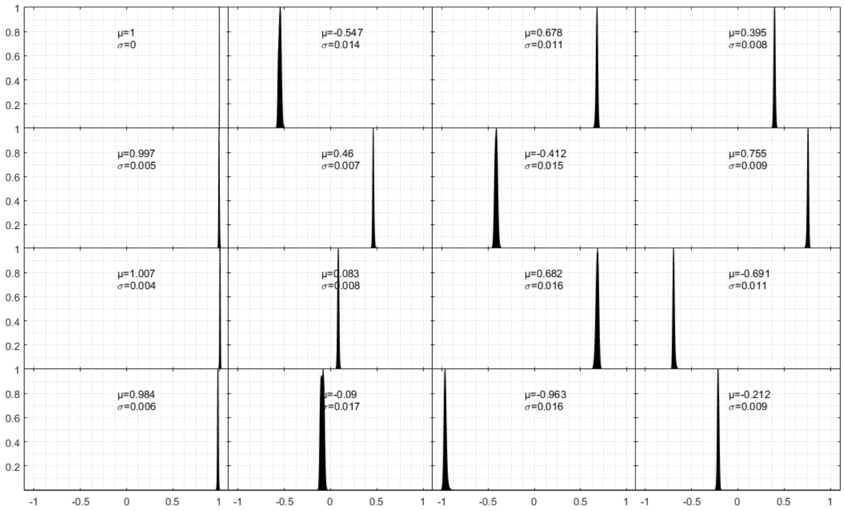

Supplementary Figure S 12: Histogram of the analysis matrix **A** displayed on Supplementary Fig. S11.

2 Calibration of the MIP

2.1 Mueller matrices of calibrated samples

Supplementary Fig. S13 shows the Mueller matrix of the sandblasted metallic plate, which is very close to the identity matrix. Mueller matrices obtained on P0, P90, and L30 components are also exhibited on Supplementary Fig. S15, Supplementary Fig. S17 and on Supplementary Fig. S19, respectively.

Corresponding histograms of these Mueller matrices are also displayed below on Supplementary Fig. S14, Supplementary Fig. S16, Supplementary Fig. S18, and on Supplementary Fig. S20, respectively. These were calculated in the Region of Interest (ROI) delineated by the dashed yellow line. This ROI corresponds to the area where the Mueller matrices of the uterine cervices have been computed. Mean and standard deviation values of all histograms have been precised by the letters "μ" and "σ", respectively. Maximum discrepancies between the theoretical red-colored mean values μ of these histograms, and the experimental ones in black were found to be lower than 0.026.

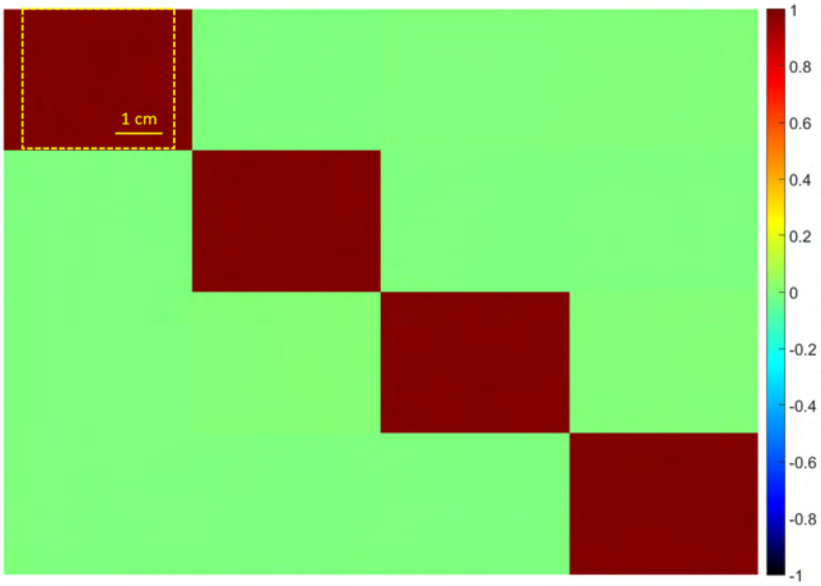

Supplementary Figure S 13: Mueller matrix of the metallic sandblasted plate.

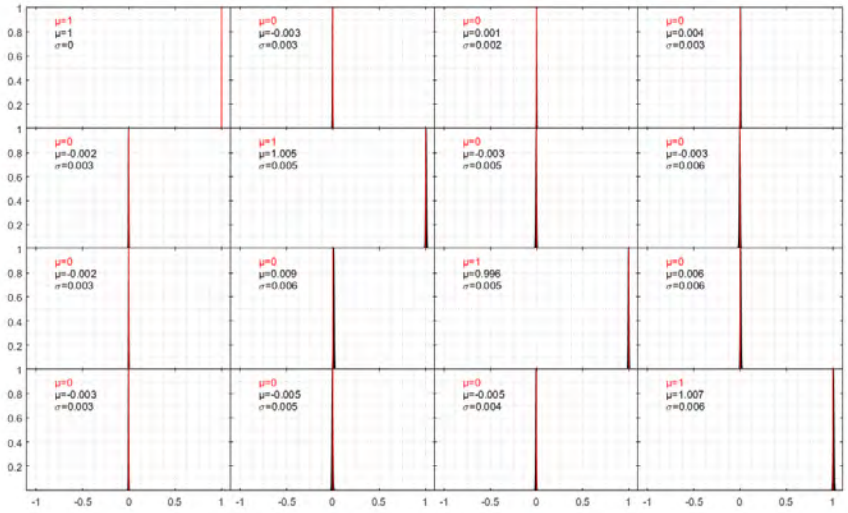

Supplementary Figure S 14: Histogram of the Mueller matrix displayed on Supplementary Fig. S13.

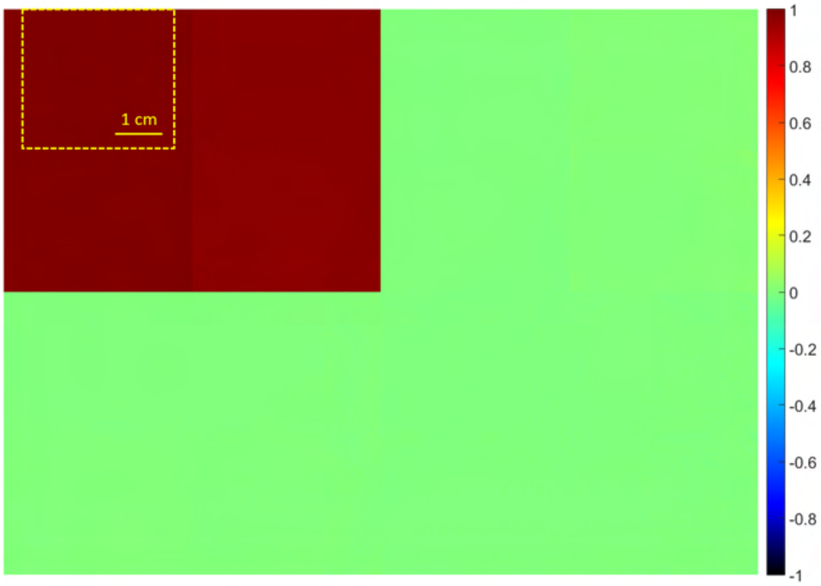

Supplementary Figure S 15: Mueller matrix of P0 component (linear polarizer at 0°).

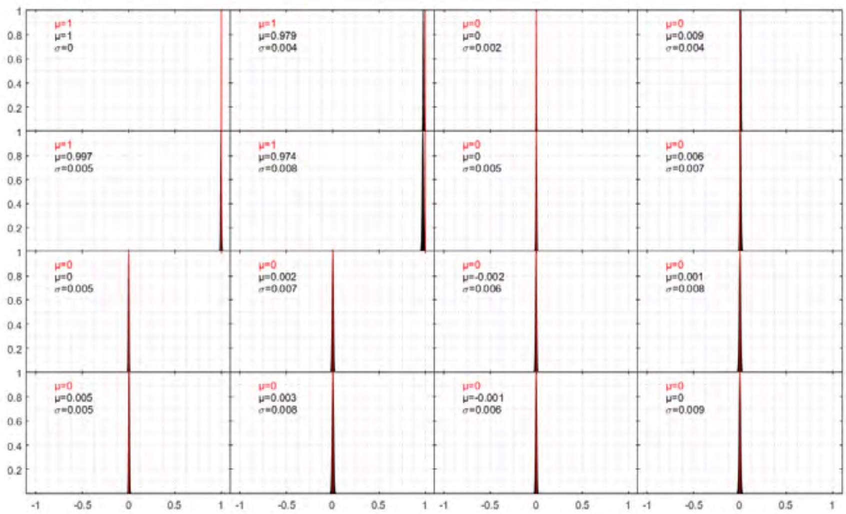

Supplementary Figure S 16: Histogram of the Mueller matrix displayed on Supplementary Fig. S15.

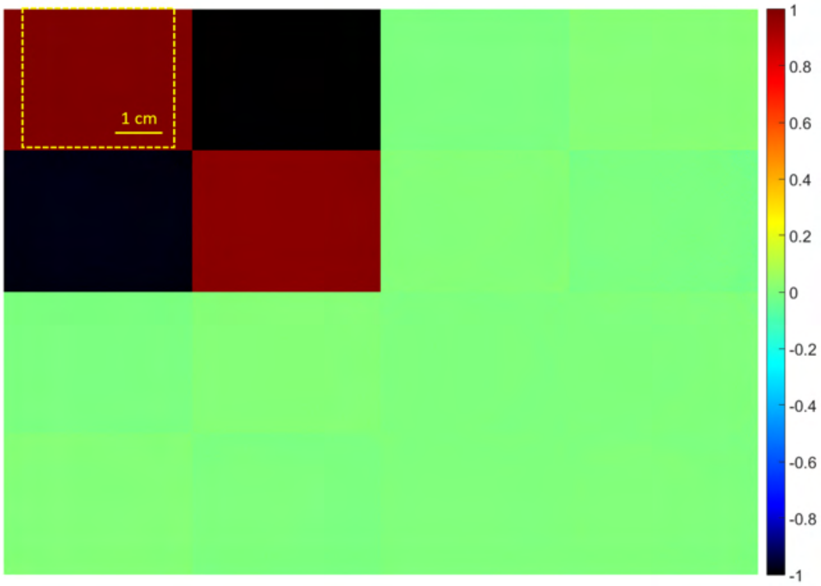

Supplementary Figure S 17: Mueller matrix of P90 component (linear polarizer at 90°).

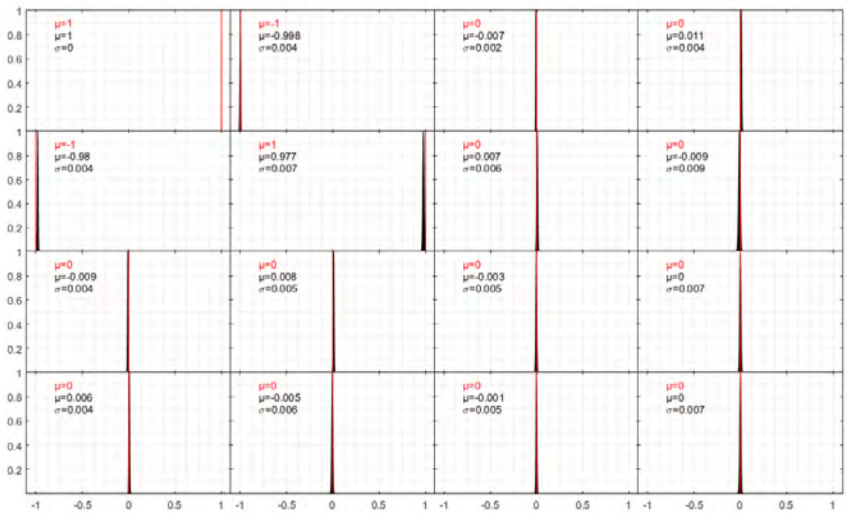

Supplementary Figure S 18: Histogram of the Mueller matrix displayed on Supplementary Fig. S17.

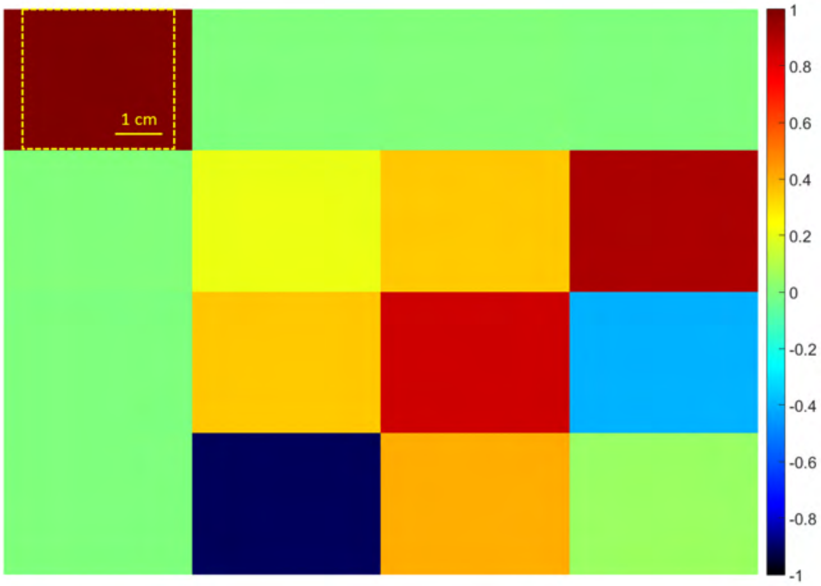

Supplementary Figure S 19: Mueller matrix of L30 component (quarter waveplate @ 532 nm at 32.9°).

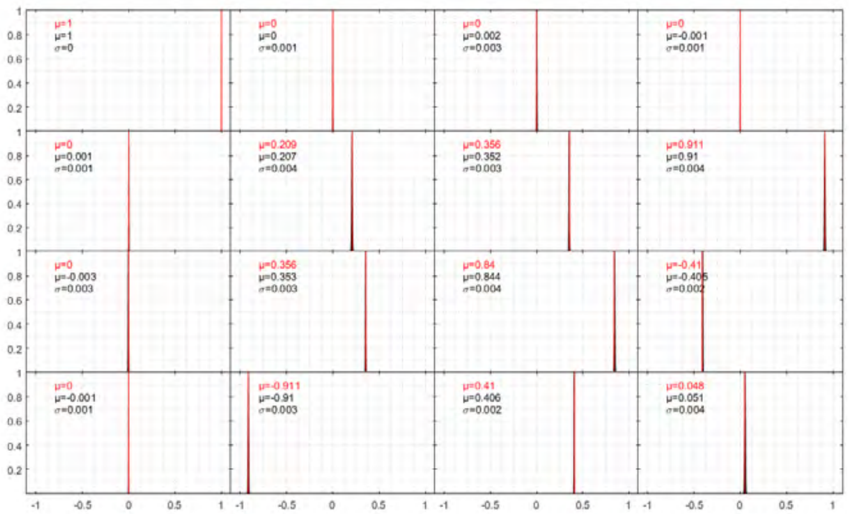

Supplementary Figure S 20: Histogram of the Mueller matrix displayed on Supplementary Fig. S19.

2.2 Modulation and analysis matrices

Supplementary Fig. S21 and Supplementary Fig. S23 show the modulation and analysis matrices **W** and **A** respectively. Corresponding histograms of these matrices are also displayed below on Supplementary Fig. S22 and Supplementary Fig. S24, respectively.

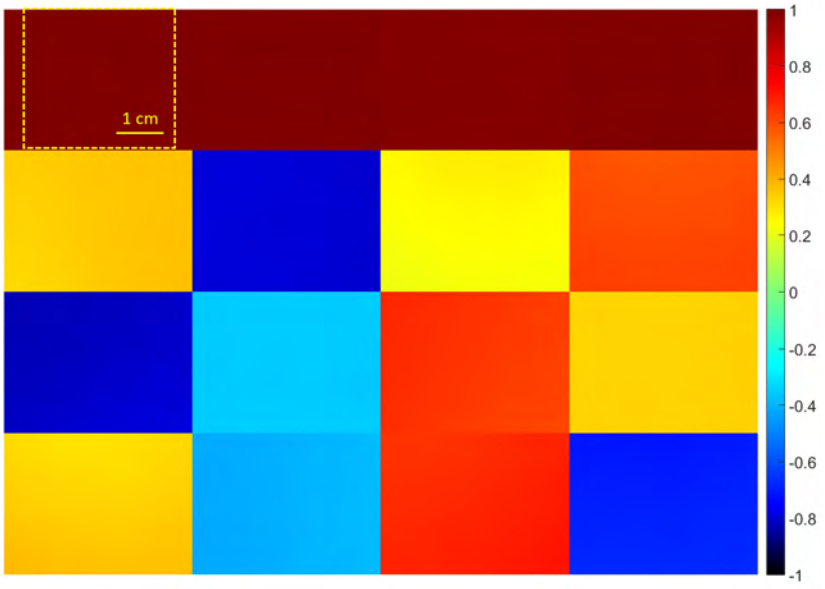

Supplementary Figure S 21: Modulation matrix **W**.

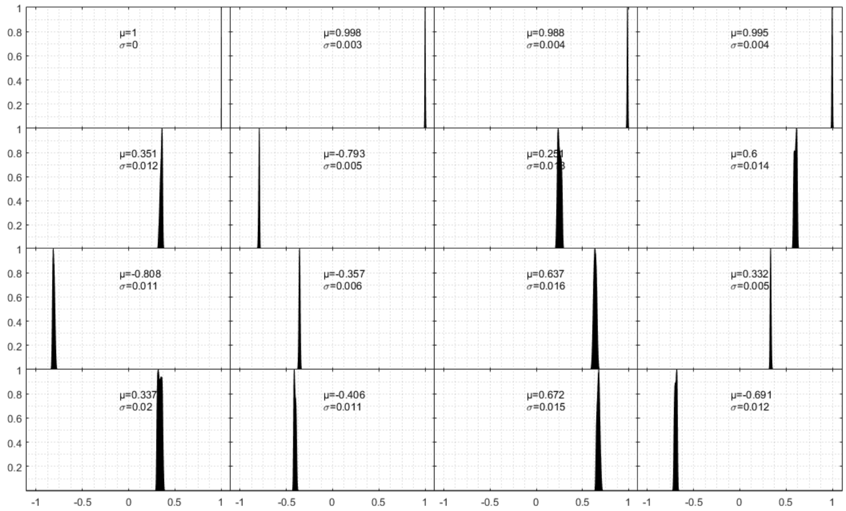

Supplementary Figure S 22: Histogram of the modulation matrix **W** displayed on Supplementary Fig. S21.

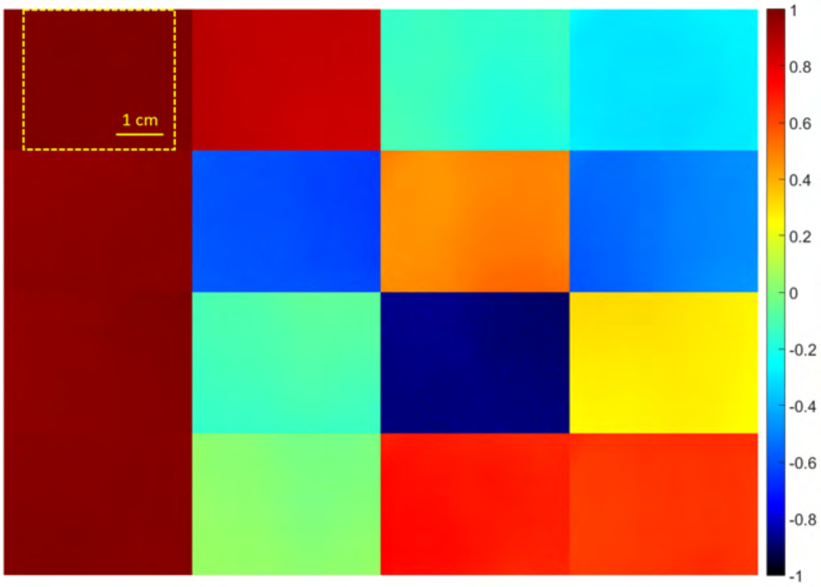

Supplementary Figure S 23: Analysis matrix **A**.

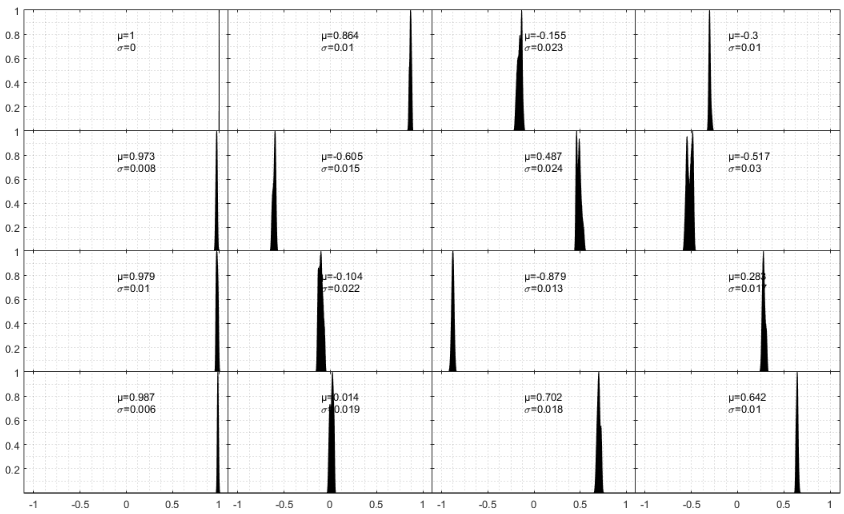

Supplementary Figure S 24: Histogram of the analysis matrix **A** displayed on Supplementary Fig. S23.
